# Individuals With Higher CD4/CD8 Ratio Exhibit Increased Risk of Acute Respiratory Distress Syndrome and In-Hospital Mortality During Acute SARS-CoV-2 Infection

**DOI:** 10.3389/fmed.2022.924267

**Published:** 2022-06-23

**Authors:** Ana Pascual-Dapena, Juan José Chillaron, Gemma Llauradó, Isabel Arnau-Barres, Juana Flores, Inmaculada Lopez-Montesinos, Luisa Sorlí, Juan Luis Martínez-Pérez, Silvia Gómez-Zorrilla, Juan Du, Natalia García-Giralt, Robert Güerri-Fernández

**Affiliations:** ^1^Medicine and Life Sciences Department, Pompeu Fabra University, Barcelona, Spain; ^2^Departament de Medicina, Universitat Autonoma de Barcelona, Barcelona, Spain; ^3^Endocrinology Department, Hospital del Mar Institute of Medical Research, Barcelona, Spain; ^4^Geriatrics Department, Hospital del Mar Institute of Medical Research, Barcelona, Spain; ^5^Infectious Diseases Department, Hospital del Mar Institute of Medical Research, Barcelona, Spain; ^6^Centro de Investigación Biomédica en Red en Enfermedades Infecciosas, Ciberinfecc, Instituto de Salud Carlos III, Madrid, Spain

**Keywords:** SARS-CoV-2, prognose, mortality, CD4/CD8 ratio, ARDS

## Abstract

**Background:**

CD4/CD8 ratio has been used as a quantitative prognostic risk factor in patients with viral infections. This study aims to assess the association between in-hospital mortality and at admission CD4/CD8 ratio among individuals with acute SARS-CoV-2 infection.

**Methods:**

This is a longitudinal cohort study with data of all consecutive patients admitted to the COVID-19 unit at Hospital del Mar, Barcelona, Spain for ≥48 h between March to May 2020. The CD4+ CD8+ T-cell subset differentiation was assessed by flow cytometry at admission as well as a complete blood test. Patients were classified according to CD4/CD8 ratio tertiles. The primary outcome was in-hospital mortality and the secondary outcome was acute respiratory distress (ARDS).

**Results:**

A total of 338 patients were included in the cohort. A high CD4/CD8 ratio (third tertile) was associated with a higher in-hospital mortality [adjusted Cox model hazard ratio (HR) 4.68 (95%CI 1.56–14.04, *p* = 0.006), reference: second tertile HR 1]. Similarly, a high CD4/CD8 ratio (third tertile) was associated with a higher incidence of ARDS [adjusted logistic regression model OR 1.97 (95%CI 1.11–3.55, *p* = 0.022) reference: second tertile HR 1]. There was a trend of higher in-hospital mortality and incidence of ARDS in patients within the first tertile of CD4/CD8 ratio compared with the second one, but the difference was not significant. No associations were found with total lymphocyte count or inflammatory parameters, including D-dimer.

**Conclusion:**

CD4/CD8 ratio is a prognostic factor for the severity of COVID-19, reflecting the negative impact on prognosis of those individuals whose immune response has abnormal CD8+ T-cell expansion during the early response to the infection.

## Introduction

SARS-CoV-2 has caused a world-wide pandemic, with more than 400 million confirmed cases and over 5.8 million deaths until March 2022 (1).

Since its first detection in December of 2019, several prognosis factors have been described. Some of them are directly associated with uncontrolled immune response to the virus leading to a hyperinflammatory status, and some other ones such as hypoalbuminemia or myocardial injury, depend on the host clinical response (2, 3). The impact of the virus in the immune system can be summarized as lymphopenia, which has been widely reported as a notable aspect of SARS-CoV-2 infection. This is common to other respiratory viral infections, including influenza A H3N2 virus or human rhinovirus (4, 5). However, lymphopenia associated with SARS-CoV-2 infection is more intense, it lasts longer (5, 6) and it seems to be more selective in T cell lineages, with a higher impact on CD8+ T cells (7, 8). Notwithstanding, a larger wide-spread lymphopenia involving CD4+ T cells, CD8+ T cells, B cells and natural killer cells has also been reported (9, 10).

The CD4/CD8 ratio has shown relevance in chronic viral infections such as HIV infection, in which the CD4/CD8 ratio has been reported as a quantitative outcome reflecting the critical role of both T-cells subsets in the HIV pathogenesis or disease progression. Examination of CD4/CD8 ratio as a quantitative trait can be important to patient care as it might be used as a prognostic risk factor (11–13).

This study aims to assess the association between in-hospital mortality and at admission CD4/CD8 ratio in individuals with acute SARS-CoV-2 infection, to elucidate if a T-cell subset disbalance might be behind an abnormal response of the host against the virus.

## Materials and Methods

### Study Design and Patient Selection

A longitudinal cohort study where data of all consecutive patients admitted to the COVID-19 unit at Hospital del Mar, Barcelona, Spain for ≥48 h between March to May 2020 were collected. The study procedures have previously been described (3, 14). As per protocol, the differentiation of CD4+ CD8+ T-cell subset was assessed in all admitted patients by flow cytometry at admission (day 1 of hospitalization), as well as a complete blood test.

The single-cell suspensions were stained with Aqua-viability dye and PacBlue-anti-CD3 and ECD-anti-CD4 from Beckman-Coulter (Brea, CA, United States); and APC-H7-anti-CD8 from Becton-Dickinson (San Jose, CA, United States) according to manufacturers’ recommendations. Fluorescence-activated cell sorter analysis was performed on a custom Becton-Dickinson LSR II flow cytometer used for data acquisition and analyzed with FlowJo (TreeStar, Ashland, OR, United States). A representative example of the gating strategy for the lymphocyte subsets is shown in Supplementary Figure 1.

Patients were classified according to CD4/CD8 ratio tertiles. The primary outcome was in-hospital mortality and the secondary outcome was acute respiratory distress syndrome (ARDS) defined as a PaO_2_-to-FiO_2_ ratio < 200 and compatible alveolar X-Ray infiltrates.

### Ethics Considerations

The Institutional Ethics Committee of Hospital del Mar of Barcelona approved the study and due to the nature of the retrospective data review and the emergent situation derived from the SARS-coV-2 pandemic, waived the need for informed consent from individual patients (CEIm 2020/9352).

### Statistical Analysis

Continuous variables are expressed as mean and standard deviation or the median and interquartile range (IQR). Qualitative variables are presented as frequencies (percentages). Normality for baseline characteristics was evaluated by Shapiro–Wilk normality test. Quantitative data was analyzed by Kruskal–Wallis test and Dunn’s test or Wilcoxon rank-sum (Mann–Whitney) test, and qualitative data was analyzed by chi-square test or Fisher exact test, as required. Appropriate coefficient tests were used for correlation among various continuous variables.

The association between the CD4/CD8 ratio and its tertiles and the primary outcome (in-hospital mortality) was assessed with unadjusted/adjusted Cox proportional hazards models. The reference group was the second tertile of CD4/CD8 ratio, for representing the most physiological range of values. Results are expressed as hazard ratio (HR) [95% confidence interval (CI)]. The same model was explored with tertiles of CD4+ T-cells and CD8+ T-cells. The association between the secondary end points and the CD4/CD8 ratio and its tertiles was assessed by logistic regression. The area under curve (AUC)-receiver operating characteristics (ROC) curve was obtained for the CD4/CD8 ratio analyzed as a continuous variable.

The level of significance in this study was set at a *p* ≤ 0.05 and CI of 95%. All statistical analyses were performed using STATA/MP V.14.

## Results

A total of 388 individuals admitted to COVID-19 unit were included in the eligibility review, 210 (54%) males and 178 (46%) females. Median age was 63 years (IQR 52–75). Patients were divided into three groups according to the CD4/CD8 ratio tertile. Baseline characteristics of each group are shown in Table 1. Tertile 2 which encompasses the more physiological range of values (1.49–2.41; 15) was considered as reference for comparisons. This group showed the lowest rate of in-hospital mortality, the lowest intensive care unit admissions and the lowest acute respiratory distress syndrome (ARDS) incidence (Table 1).

**TABLE 1:** Baseline characteristics and the comparison according to the three different CD4/CD8 T-cell ratio tertiles.

	Overall	CD4/CD8 T-cell ratio			
		First tertile (≤1.494)	second tertile (1.495–2.413)	Third tertile (≥2.414)	*p*-value
*n*	388	130	129	129	
Age, median (IQR)	63 (52–75)	65 (53–77)	62 (50–74)	64 (53–73)	0.409
Male, *n* (%)	210 (54%)	80 (62%)	66 (51%)	64 (49%)	0.082
Current smoker, *n* (%)	28 (7%)	10 (7%)	6 (4.6%)	12 (9.3%)	0.347
Days of symptoms, median (IQR)	7 (4–9)	7 (5–9)	7 (4–9)	7 (5–9)	0.732
In-hospital stay, days, median (IQR)	9 (6–16)	9 (6–15)	9 (5–16)	9 (6.5–17)	0.843
**Comorbidities, *n* (%)**					
Hypertension	215 (55%)	69 (53%)	66 (51%)	80 (62%)	0.173
Diabetes mellitus	95 (24%)	32 (24%)	36 (28%)	27 (21%)	0.427
Cardiovascular disease	45 (11.6%)	12 (9.2%)	13 (10%)	20 (15.5%)	0.261
Chronic respiratory disease	34 (8.7%)	11 (8.4%)	13 (10%)	10 (7.7%)	0.812
Chronic kidney disease	120 (30%)	50 (38%)	38 (29.5%)	32 (24.5%)	0.052
Chronic liver disease	30 (7.7%)	15 (11.5%)	6 (4.6%)	9 (7%)	0.134
Immune condition	16 (4%)	4 (3%)	5 (4%)	7 (5%)	0.411
**Charlson comorbidity index**					
No comorbidity	171 (44%)	58 (44%)	54 (42%)	59 (45%)	0.933
Medium-low (1–2)	125 (32%)	35 (27%)	51 (39.5%)	39 (30%)	0.083
High (≥3)	92 (24%)	37 (28.5%)	23 (18%)	32 (25%)	0.118
**Clinical markers at onset, median (IQR)**					
C-Reactive protein mg/dl	7 (3.1–15.6)	6.8 (3.7–13.4)	6.1 (2.7–14.5)	7.8 (2.8–19.2)	0.772
IL-6 pg/ml	40 (13–83)	46.6 (19.9–75.7)	33.8 (13.2–83.8)	39.3 (10–92)	0.928
D-Dimer UI/l	765 (460–1,330)	870 (520–1,550)	650 (390–1,020)*	825 (460–1,520) ^‡^	0.018
Creatinin mg/dl	0.93 (0.73–1.12)	0.94 (0.74–1.14)	0.9 (0.73–1.07)	0.93 (0.73–1.14)	0.329
**Hemogram, median (IQR)**					
Lymphocyte 10^6^ count/ml	1.03 (0.75–1.39)	1.04 (0.73–1.45)	1.09 (0.74–1.41)	0.98 (0.77–1.31)	0.679
CD4+ T-cell (10^6^ cells/ml)	0.380 (0.250–0.609)	0.285 (0.179–0.416)	0.417 (0.280–0.609)*	0.492 (0.331–0.743) ^†^ ^‡^	<0.001
CD8+ T-cell (10^6^ cells/ml)	0.199 (0.123–0.345)	0.295 (0.181–0.435)	0.211 (0.150–0.339)*	0.127 (0.079–0.191) ^†^ ^‡^	<0.001
**Severity parameters, median (IQR)**					
MEWS	2 (1–3)	2 (1–3)	2 (1–3)	2 (2–3)	0.401
PaO_2_-to-FiO_2_ ratio,	208 (112–310)	210 (132–317)	265 (92–316)	171 (108–278)	0.249
CURB-65	1 (0–2)	1 (0–2)	1 (0–2)	1 (0–2)	0.157
**Outcomes, *n* (%)**					
In-hospital Mortality	33 (8.5%)	12 (9%)	5 (4%)	16 (12%)^‡^	0.050
ARDS	99 (25%)	35 (27%)	24 (19%)	40 (31%)	0.078
ICU-Admission	85 (21%)	29 (22%)	25 (19%)	31 (24%)	0.674

*Data are given as median (IQR) or n (%) unless otherwise indicated. CURB-65, confusion, urea >7 mmol/L, respiratory rate $30/min, low blood pressure #90/60 mm Hg, and age 65 years; FiO_2_, fraction of inspired oxygen; MEWS, Modified Early Warning Score; PaO_2_, arterial partial pressure of oxygen.*

*P < 0.05 for second tertile compared with first tertile.

*^†^P < 0.05 for third tertile compared with first tertile.*

*^‡^P < 0.05 for third tertile compared with second tertile.*

Total lymphocyte count was not different between the three tertiles (*p* = 0.679), with a median in the lower limit of reference values of a normal hemogram (median 1.03 10^6^ lymphocytes/ml, IQR 0.75–1.39), showing a common tendency to lymphopenia in all groups. The first tertile showed the lowest CD4+ T-cell count and the third tertile showed the lowest CD8+ T-cell count, suggesting that the abnormal CD4/CD8 ratio was due to depletion of CD4+ T-cells or lack of expansion of CD8+ T-cells, respectively, and not because of an anomalous expansion of one of the cell-types. No significant differences in inflammatory markers were found among groups, except for D-dimer levels which were higher in first and third tertiles (*p* = 0.018), showing an increased underlying infection activity compared to the reference second tertile. However, these differences were not sustained after multivariate adjustment.

Additionally, no significant differences were observed among tertile groups in the severity scores at admission, nor in the main comorbidities. In spite of not being different across all tertiles, chronic kidney disease was significantly more prevalent in patients with lower ratios (first tertile) when compared to the highest ratios (third tertile; *p* = 0.018), but showed no differences when compared to the reference tertile.

We found a significant association between CD4/CD8 ratio and in-hospital mortality [HR1.08 (95%CI: 1.03–1.41); *p* = 0.002]. Each unit increase in the ratio was associated with an 8% increase in mortality. Thus, higher CD4/CD8 ratios (third tertile) was significantly associated with increased risk of in-hospital mortality [HR 4.42 (95% CI: 1.36–12.55); *p* = 0.005] respect to second tertile (Table 2). We also found this trend in lower ratios (first tertile), but without statistical significance as compared to the reference [HR 2.69 (95% CI: 0.94–7.69); *p* = 0.064]. This association presents a comprehensive Kaplan–Meier log rank of 8.75 (*p* = 0.012). After adjusting by age, sex, comorbidity, and severity of the episode, the third tertile remained significantly associated with in-hospital mortality [HR 4.68 (1.56–14.04); *p* = 0.006] (Table 2). No association was found with mortality and total lymphocyte count or inflammatory parameters, including D-dimer. No global differences between CD4/CD8 ratio and the incidence of ARDS were found (*p* = 0.078). However, when analyzing across tertiles, the third tertile with higher CD4/CD8 ratio showed an increased incidence of ARDS after adjusting by age, sex and comorbidity [OR 1.97 (95% CI: 1.11–3.55); *p* = 0.022] than the reference group. There was no association between ARDS and lower ratios (*p* = 0.131).

**TABLE 2:** Adjusted models for in-hospital mortality and ARDS risk according to the CD4/CD8 T-cell ratio tertiles.

In-hospital mortality	Hazard ratio	CI (95%)		*p*-value
First tertile (Ref: 1, second tertile)	2.16	0.71	6.58	0.175
Third tertile (Ref: 1, second tertile)	4.68	1.56	14.04	0.006

**ARDS**	**Odds ratio**	**CI (95%)**		***p*-value**

First tertile (Ref: 1, second tertile)	1.58	0.87	2.87	0.131
Third tertile (Ref: 1, second tertile)	1.97	1.11	3.55	0.022

*ARDS: Acute respiratory distress syndrome.*

*Adjusted models. For In-hospital mortality we used a Cox Proportional Hazards model, adjusted for age, sex, comorbidity, and severity of the episode. For ARDS we applied a logistic regression model adjusted for age, sex, comorbidity, and severity.*

**FIGURE 1:**
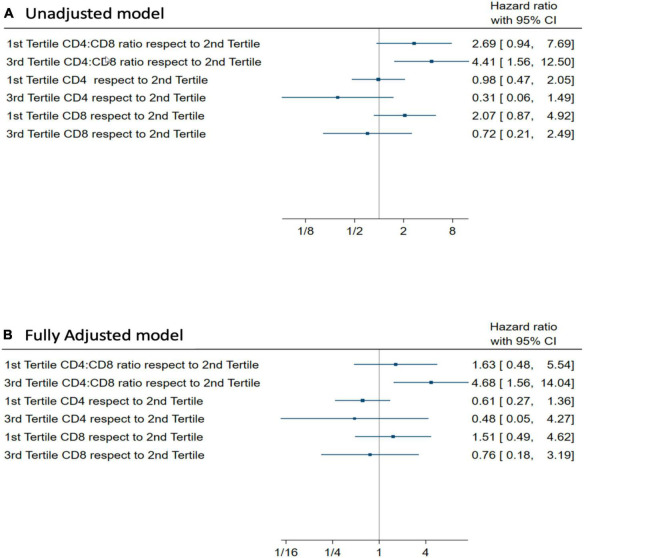
**(A)** Unadjusted Hazard ratio for In-hospital mortality (Cox Proportional Hazards model) for CD4/CD8 ratio tertiles, CD4 tertiles, and CD8 tertiles. **(B)** Adjusted Hazard ratio by age, sex, comorbidity, and severity for in-hospital mortality (Cox Proportional Hazards model) for CD4/CD8 ratio tertiles, CD4 tertiles, and CD8 tertiles.

Several sensitivity analyses were performed, creating models that controlled for specific comorbidities like chronic kidney disease, inflammatory markers, as well as creating an alternative division of groups (considering standard measures of CD4/CD8 ratio: <1.0, 1.0–2.5, >2.5, instead of tertiles). All analyzes yielded similar findings that are in favor of those already presented (data not shown). In an alternative exploratory model with the same adjustment but considering CD4 and CD8 tertiles, no association was found with in-hospital mortality (Figure 1).

**FIGURE 2:**
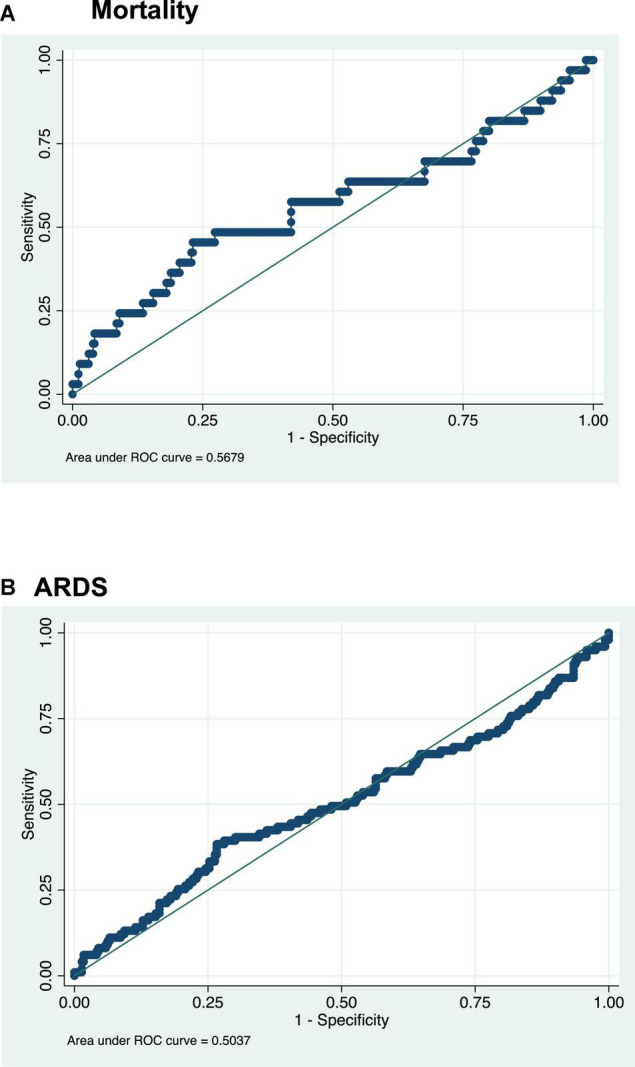
ROC curve for the CD4/CD8 ratio: **(A)** The best cutoff for mortality discrimination is ≥ 2.027 Sensitivity: 57.58% Specificity: 58.03%; **(B)** the best cutoff for ARDS discrimination is ≥ 1.945 Sensitivity: 48.48% Specificity: 53.98%.

Receiver Operating Characteristics curves and AUCs were used to assess the discriminative accuracy of the CD4/CD8 ratio on mortality (Figure 2A) and ARDS (Figure 2B). In this case, the CD4/CD8 ratio was analyzed as a continuous variable. The AUC (95% CI) for discriminating mortality was 0.56 (0.44–0.68), standard error = 0.061. Using the best cut-off point of 2.027 in the CD4/CD8 ratio, the sensitivity was 57.6% with a specificity of 58.03%. The AUC (95% CI) for discriminating ARDS was 0.50 (0.43–0.57), standard error = 0.036, hence the model using lineal values of the CD4/CD8 ratio has no discrimination capacity to distinguish between patients with or without ARDS.

## Discussion

We report an association between the CD4/CD8 ratio and in-hospital mortality due to acute SARS-CoV-2 infection, regardeless of total lymphocyte count. Higher ratios (third tertile), which demonstrated lack of CD8+ T cell expansion, were significantly associated with mortality, but also lower ratios (first tertile) showed a trend to worse prognosis.

An abnormal CD4/CD8 ratio is often viewed as clinically relevant (16–18), it rarely measures below 1.0 or above 2.5 (15) and its disbalance by excess or defect always offers insights about immune malfunction. There is evidence that the CD4/CD8 ratio is genetically controlled in healthy humans (15, 19) and in the case of sarcoidosis, for example, a higher CD4+/CD8+ ratio is found in patients who carry HLA-DRB1*03 (19).

Indeed, the CD4/CD8 ratio has been used clinically in different scenarios: For example in the diagnosis of sarcoidosis and in chronic granulomatours diseases where a depletion in CD8+ T-cells leading to a higher CD4/CD8 ratio seems to be protective to worse disease presentations (19). Conversely, in HIV infection, the CD4/CD8 ratio is abnormally low (20). This shows on the one hand, the damage that the infection induces in the CD4+ T-cell compartment, and on the other hand, the restoration of the immune system after starting antiretroviral treatment, which sometimes restores CD4+ T-cells correctly but favors an over-expansion of CD8+ T-cell compartment (12, 21, 22). Thus, a CD4/CD8 ratio below 1 in individuals with HIV under antiretroviral therapy shows an incomplete and abnormal reconstitution of the immune system (12, 23, 24). These low ratios have been associated with worse outcomes like increased cardiovascular events, long-term complications, and all-cause mortality (18). Hence, there are clinical scenarios in which a depletion in CD8+ T-cells can be beneficial, and others where an expansion can be detrimental.

A particular scenario occurs in SARS-CoV-2 infection where, as we report, the highest tertile of CD4/CD8 ratio (with values > 2.4) is associated with a higher risk in-hospital death during the acute infection. There is also a trend in the case of the lower tertile of the CD4/CD8 ratio which could be taken into consideration.

In our cohort, individuals with higher CD4/CD8 ratio levels had lower CD8+ T-cell levels, showing an inadequate expansion of CD8+ cytotoxic T-cells, while those with lower CD4/CD8 ratio levels had lower CD4+ T-cell levels (not an abnormal expansion of CD8+ T-cells).

Therefore, the absence of an adequate expansion of CD8+ T-cells, leading to higher CD4/CD8 ratios, has a deleterious impact on the prognosis of the infection in terms of in-hospital mortality but also in ARDS incidence (13, 25).

This highlights the determining role of cytotoxic cellular immunity in the response in the acute phase of infection, setting its importance as keystone for a first line defense against SARS-CoV-2 infection. Although this fact has been reported by some authors, the evidence remained inconclusive (26, 27) or was based in small populations (28).

We can infer that an impaired CD8+ T-cell response with low cell clonal expansion, may lead to a poorer infection control and consequently worse prognosis, as opposed to a high and robust clonal expansion of this subset of T-cell that may be associated with milder forms of the disease (29, 30). In fact, an early development of a cytotoxic CD8+ T cell response, typically observed within 7 days of symptoms and peaking at 14 days, is correlated with effective viral clearance (7) and mild disease (31). In this line, Zuani et al. studied the T-cell subset composition in the peripheral blood of COVID-19 survivors and non-survivors (11). Analyzing CD8+ T-cell subpopulations, they observed lower counts in memory cells and effector memory cells re-expressing CD45RA (temra) among non-survivors with a lower absolute CD8+ T-cell count (11).

Notably, our patients with lower CD8 levels leading to a higher CD4/CD8 ratio did not present a more severe acute presentation of infection nor more inflammation at admission to the hospital when comparing to the other groups. It reflects the important role of the CD8+ T-cell subset in controlling the infection in its early stages, before an hyperinflammatory state has been established.

However, we also found a trend to a worse prognosis among the individuals in the lowest tertile of CD4/CD8 ratio. Similarly to what happens in HIV infection inadequate and persistent expansion of CD8+ T-cells can lead to an excessive uncontrolled immune response that can also affect the prognosis of the SARS-CoV-2 infection.

In fact, individuals with the lowest or highest CD4/CD8 ratio have worse prognosis than individuals within the second tertile. This could be related to the fact that patients within the lowest tertile also have a trend increased mortality. Hence, the risk of a poorer prognosis is determined by the fact of having an unbalanced CD4/CD8 out of the normal range.

This study has some limitations that must be stated. Firstly, it is a single-center study, with the consequent limited population. Also, the retrospective nature of the study does not allow us to establish causality between the CD4/CD8 ratio and the outcome. In addition, only CD4+ or CD8 T-cells global count were analyzed. Further studies to evaluate how naïve, effector memory cells and other subpopulations are associated with worse prognosis, pinpointing the underlaying mechanisms for the depletion of CD8+ Tcells and broadening our findings.

This study also presents some strengths. Importantly, samples are consistently collected at admission for all patients, therefore not generating bias and being a good representation of patients who need hospitalization due to COVID-19. Likewise, the healthcare was provided under the same guidelines by the same group of healthcare providers, eliminating variability that could lead to difference in outcomes.

In conclusion, CD4/CD8 ratio is a prognostic factor for acute SARS-CoV-2 infection independent of CD4 or CD8 alone, reflecting the negative impact on prognosis of those individuals whose immune response is disbalanced with an abnormal CD8+ T-cell depletion during the early response to the infection. This is different to other viral infections such as HIV, for which persistently low CD4/CD8 ratio is associated with worse outcome and increased risk of non-AIDS (18), or systemic diseases like sarcoidosis, for which lack of expansion and high ratios is advantageous.

## Data Availability Statement

Some or all datasets generated during and/or analyzed during the current study are not publicly available but could be available from the corresponding author on reasonable request.

## Ethics Statement

The studies involving human participants were reviewed and approved by Ethics Committee PsMar CEIm 2020/9352. Written informed consent for participation was not required for this study in accordance with the national legislation and the institutional requirements.

## Author Contributions

RG-F, AP-D, and JC conceptualized and conducted the study. AP-D, RG-F, JC, and NG-G revised methodology and did data analysis. IA-B, IL-M, JL, SG-Z, GL, and JD contributed with recruitment and data curation. All authors have revised and edited the final manuscript.

## Conflict of Interest

The authors declare that the research was conducted in the absence of any commercial or financial relationships that could be construed as a potential conflict of interest.

## Publisher’s Note

All claims expressed in this article are solely those of the authors and do not necessarily represent those of their affiliated organizations, or those of the publisher, the editors and the reviewers. Any product that may be evaluated in this article, or claim that may be made by its manufacturer, is not guaranteed or endorsed by the publisher.
