# Supplementary figures and images for: Individuals With Higher CD4/CD8 Ratio Exhibit Increased Risk of Acute Respiratory Distress Syndrome and In-Hospital Mortality During Acute SARS-CoV-2 Infection
Source: Front Med (Lausanne). 2022 Jun 23;9:924267. doi: 10.3389/fmed.2022.924267 (PMC9260079; doi:10.3389/fmed.2022.924267)

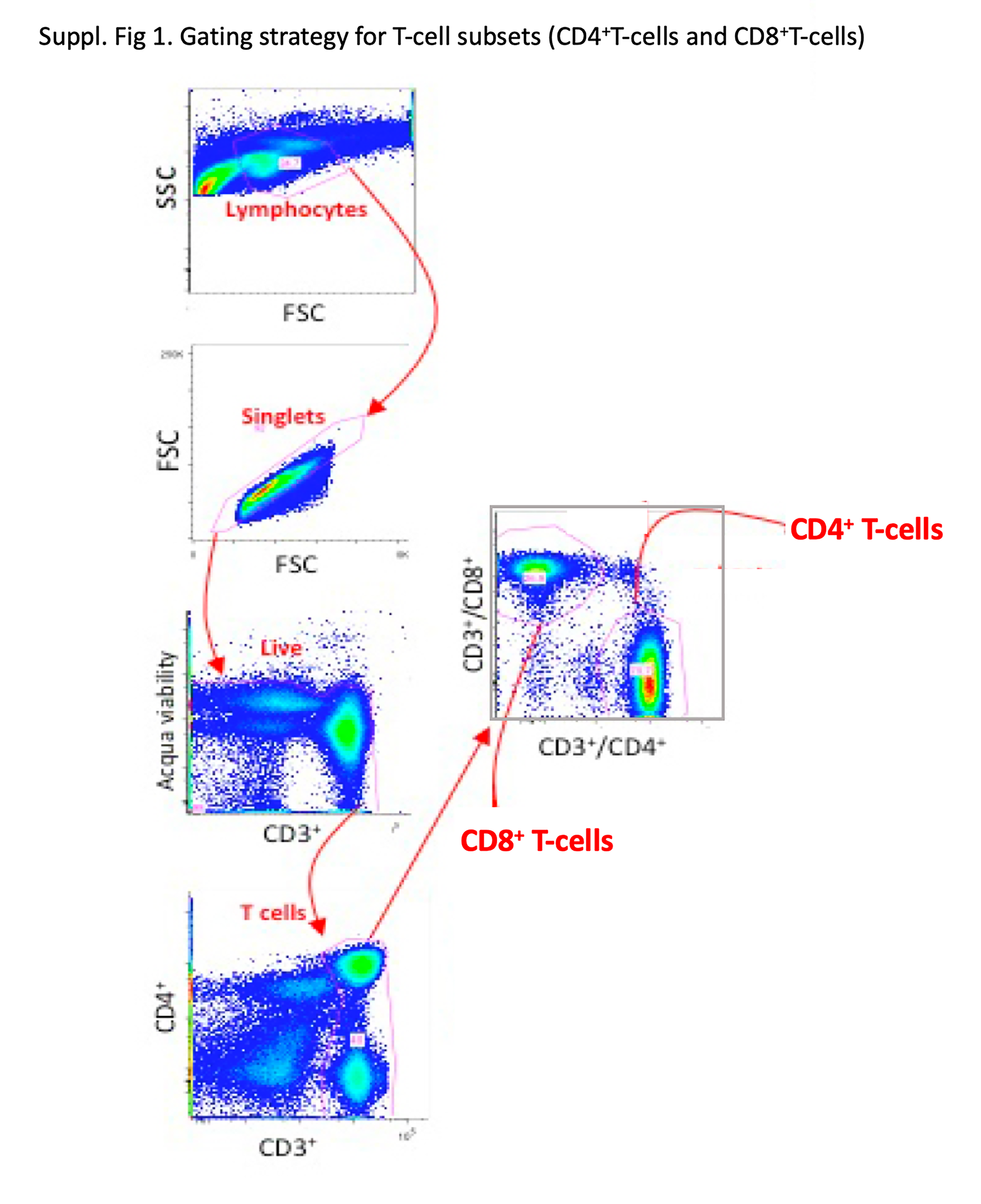

Supplement: Supplementary file 1 [file Image_1.TIFF]
